# A micro-sociological approach to prolonged grief disorder: identification and measurement of simpatico, a novel interpersonal risk factor

**DOI:** 10.1192/bjo.2026.11028

**Published:** 2026-04-24

**Authors:** Gizem Cesur-Soysal, Madeleine M. Hardt, Kirsten V. Smith, Paul K. Maciejewski, Holly Gwen Prigerson

**Affiliations:** Department of Psychology, Istanbul Medipol University – Kavacık Campus, Türkiye; Department of Radiology, Weill Cornell Medicine, Cornell Center for Research on End-of-Life Care, New York, USA; Department of Experimental Psychology, The Oxford Centre for Anxiety Disorders and Trauma, University of Oxford, UK

**Keywords:** Bereavement, prolonged grief, simpatico, relationships, social support

## Abstract

**Background:**

Prolonged grief disorder (PGD) is characterised by persistent yearning and intense emotional pain, and is often accompanied by identity disruption and social withdrawal. Research has identified various PGD risk factors; however, limited research has examined how relationship to the deceased relates to PGD risk.

**Aims:**

This study introduces ‘simpatico’, a novel construct assessing a mourner’s perceived similarity and connection to the deceased as a risk factor for PGD. Grounded in the micro-sociological theory of bereavement, the study hypothesises that simpatico relationships heighten PGD risk because of the particular social deprivations their absence creates.

**Method:**

This cross-sectional study was conducted in Turkey via online surveys with 400 bereaved. Data were analysed using exploratory and confirmatory factor analyses, as well as correlation- and regression-based analyses.

**Results:**

A nine-item Simpatico Scale was validated within a Turkish bereaved adult sample (*N* = 400), demonstrating good internal consistency (Cronbach’s *α* = 0.90). Regression analyses revealed that elevated simpatico scores, particularly felt similarities with the deceased, were significantly associated with PGD symptom severity (*β* = 0.31, *p* < 0.001), even when controlling for demographic, cause of death, relationship to deceased and social support variables.

**Conclusions:**

Results identify simpatico as a new, particularly influential, interpersonal risk factor for PGD. Further, according to the micro-sociological theory, results suggest that promotion of simpatico relationships among bereaved persons may compensate for the social deprivations resulting from simpatico relationship losses. In these ways, this study identifies mourners at risk and suggests promising ways to intervene to reduce mourners’ risk of PGD.

The micro-sociological theory of adjustment to loss^1^ is based on the conceptualisation of bereavement as a social loss; that is, the loss of an important other person. It proposes that individuals who survive the loss of someone who provided a sense of meaning, purpose and shared/aligned identity experience significant social deprivations in the wake of that social loss. In light of this theory it is postulated that social deprivations or voids created by the death of someone with whom the bereaved survivor felt understood or similar to (i.e. a ‘simpatico’ other) are expected to heighten the mourner’s risk of prolonged grief disorder (PGD).^1^

PGD, a recently recognised diagnosis in the DSM-5-TR as well as the ICD-11,^2–4^ is characterised by persistent yearning, intense emotional pain, difficulty accepting the loss, emotional numbness, identity disruption, social withdrawal and a pervasive sense of meaninglessness. PGD is a risk for diverse and detrimental outcomes such as suicidal ideation,^2^ high blood pressure and cancer.^5^ Consequently, there is a public health interest in identifying influences and targets of intervention to reduce a mourner’s risk. Adult prevalence estimates of PGD vary by diagnostic criteria and loss characteristics, ranging from 1.5 to 15.3% in representative bereaved samples using ICD-11 criteria,^6^ and around 7–10% in general bereaved populations assessed with DSM-5-TR or mixed criteria,^3^ with higher estimates when screening tools rather than full diagnostic interviews are used; rates may rise to as high as 49% following violent or traumatic losses.^7^ Prevalence in bereaved children and adolescents ranges from 10.4 to 32%, and often higher in samples characterised by traumatic losses, reflecting potential confounding effects.^8^

Here, we introduce the concept of ‘simpatico’, which represents the perceived interpersonal relationship of the mourner to the deceased person as a potentially important PGD risk factor. Simpatico relationships are characterised by mutual understanding and a sense of shared values and proclivities, whereby partners perceive likeness in experiences, preferences and priorities. In this sense, simpatico refers to the mourner’s perceived similarity and identification with the deceased, reflecting the extent to which the mourner experiences the other person as ‘like me’. Thus, the concept captures perceived self–other resemblance rather than emotional dependency, relational closeness or attachment security.

Previously, research has examined several relational constructs relevant to bereavement adjustment. Parkes and Weiss^9^ suggested that dependency in relationships, particularly in marital bonds, increases the risk of pathological grief.^10,11^ In addition, intimacy in the relationship with the deceased, regardless of kinship, as well as the depth of the relationship (e.g. between siblings), has been found to be positively associated with the severity of prolonged grief symptoms.^12,13^ More recently, a measure of ‘partner dependency’ has previously been developed, which was found to be significantly associated with PGD.^10,14^ Yet, dependent and simpatico relationships are conceptually distinct. Although dependency reflects emotional reliance on another person, a mourner may identify with the deceased and not necessarily be dependent on them. It is also possible for a person to be emotionally dependent on the decedent without perceiving them as ‘like me’. Simpatico refers specifically to perceived self-other similarity and identification, rather than to emotional dependency, intimacy or relational closeness.

Relatedly, ‘anaclitic’ relationships refer to dependency-based ties characterised by emotional reliance on the other, and therefore capture a form of relational dependence rather than perceived self–other resemblance. Conflict or ambivalence in the relationship with the deceased has long been considered an important risk factor for problematic adjustment to bereavement.^15–17^ More recently, research by Sekowski and Prigerson reported only a weak association between conflict and PGD, whereas a strong association was observed between relational closeness and PGD.^18^ However, simpatico is also distinct from the Freudian notion of ambivalence, which Freud proposed as a factor predisposing individuals to pathological mourning.^15^ A person may feel identified with another without feeling ambivalent or conflicted about them, and a person may also experience ambivalence toward another without perceiving them as similar.

These constructs are all relational but reflect distinct relationship types, highlighting the need to examine how they relate to one another and how they may contribute to PGD and bereavement adjustment more broadly. Although prior research has focused primarily on attachment, dependency and relational closeness, the construct of simpatico relationships with the deceased has not yet been studied. Informed by the micro-sociological theory of adjustment to loss, we hypothesise that mourners who report simpatico relationships with the deceased, characterised by a sense of mutual understanding and perceived similarity, may represent a group vulnerable to PGD.

Given that grief is shaped not only by emotional intensity, but also by interpersonal relationships both to the deceased person and those living in the mourner’s social network,^19,20^ a tool to measure simpatico fills an important gap. The newly developed Simpatico Scale (Prigerson et al, found at the Cornell Center for Research on End-of-Life Care website)^21^ offers opportunities to explore its role as a social influence on bereavement adjustment and specifically onset of PGD.

The present study has two main objectives: (a) to test the reliability and validity of the newly developed Simpatico Scale, and (b) to examine the association between simpatico relationships to the deceased person and PGD symptom severity in a sample of recently bereaved individuals.

## Hypotheses

We hypothesise that the Simpatico Scale will demonstrate acceptable psychometric properties, including reliability and construct validity; that higher simpatico levels will be positively associated with more severe PGD symptoms; and that simpatico will predict PGD symptom severity when controlling for demographic and loss and social support and other relationships to the deceased person (i.e. competing risks).

## Method

### Study design/procedures

In this cross-sectional study based in Turkey, surveys were administered online using Google Forms, from December 2024 to February 2025. All participants completed surveys in a single session. Survey completion took an average of 15 min. Ethics approval for this study was obtained from Istanbul Medipol University (approval number: 158). All participants provided written informed consent before participation.

### Participants

Inclusion criteria were having experienced a loss and being over 18 years of age. Exclusion criteria included a history of severe psychiatric disorder, (assessed by asking whether the participant had previously received a psychiatric diagnosis). A total of 17 participants were excluded because of a prior psychiatric diagnosis. Participants were recruited through two different methods. One group was recruited via announcements shared on social media platforms, targeting individuals who had experienced a loss. The other group reached with the help of university students who distributed the survey to individuals in their social circles who had experienced a loss. Students were eligible if they reported a bereavement history, involving the loss of an important other person that had occurred within the past 15 years. Students who assisted with recruitment and those who completed the survey were compensated with course credit. No significant differences were observed between recruitment methods, likely because they both targeted bereaved individuals from the general population.

The full sample was comprised of 400 participants (social media recruitment, *n* = 149; student recruitment, *n* = 251). From the full sample, two randomly selected subsamples were used for conduct of exploratory factor analysis (*n* = 194) and confirmatory factor analysis (*n* = 240). All participants (*N* = 400) were included in the regression analysis. Participant characteristics are presented in Table 1.

**Table 1:** Demographic and bereavement-related descriptive and Pearson correlation analysis with the PG-13-R (*N* = 400)

Variable	*n* (%)/mean (s.d.)	PG-13-R Pearson *r*-statistic
Gender (female = 1)^a^	319 (79.8%)	0.07
Age (years; range: 18–75)	33.7 (13.9)	0.01
Age of deceased (years; range: 0–99)	60.3 (21.5)	−0.30^***^
Time since loss (months; range: 0–144)	36.2 (29.4)	−0.09
Relationship to deceased (first-degree = 1)^b^	233 (58.3%)	0.35^***^
Cause of death (unnatural = 1)^c^	354 (88.5%)	0.26^***^

PG-13-R, Prolonged Grief-13-Revised. First-degree relatives included parent, sibling, partner and child; non-first-degree relatives included uncle, aunt, cousin, grandparent and friend; unnatural causes included accident, illness, suicide, natural disaster and human-caused events.

a. Gender (self-reported gender assigned at birth) was dummy coded as 1 = female and 0 = other.

b. Relationship to deceased person was dummy coded as 1 = first-degree relatives and 0 = non-first-degree relatives.

c. Cause of death was dummy coded as 1 = unnatural and 0 = natural causes.

*** *p* < 0.001.

### Measures

#### Grief-related measure

The 13-item Prolonged Grief-13-Revised (PG-13-R) scale was developed by Prigerson et al^2^ and validated in Turkish (details available from the author on request) to assess the presence and duration of loss (2 items), PGD symptoms (10 items rated on a 5-point Likert scale) and functional impairment (1 item). Cronbach’s *α* was 0.91 in the current sample.

#### Social support-related measures

The Lubben Social Network Scale-6 (LSNS-6) was developed by Lubben et al^22^ and adapted in Turkish by Demir and Hazer^23^ to measure social support from family and non-kin, using six self-report items. Cronbach’s *α* for total scale was 0.79 in the current sample.

The 12-item Interpersonal Support Evaluation List-Short Form (ISEL-SF) was developed by Cohen et al^24^ to assess perceived social support across 3 dimensions: appraisal (guidance), belonging (empathy, acceptance) and tangible support (financial/material aid). A Turkish version of the scale is available (details available from the author on request). Cronbach’s *α* for total score is 0.87 in the current sample.

The Interpersonal Needs Questionnaire-15 (INQ-15) was developed by Van Orden et al^25^ and adapted into Turkish by Kurşuncu and Baştemur^26^ to evaluate unmet interpersonal needs across two factors: thwarted belongingness and perceived burdensomeness to others. Cronbach’s *α* for total score was 0.88 in the current sample.

#### Deceased-relationship measures

The Simpatico Scale (Simpatico) is a nine-item survey (rated 1–5) developed by Prigerson and Maciejewski at Weill Cornell Medicine, to assess the nature of the bereaved person’s relationship with the deceased. The Turkish version of the scale is presented in the Results section of this study. Cronbach’s *α* is calculated 0.90 for the total scale.

The Quality of Relationships Inventory – Bereavement Version (QRI-B) was developed by Bottomley et al^27^ and adapted into Turkish by Keser et al^28^ to evaluate the bereaved individual’s pre-death relationship quality with the deceased, using 13 items and 2 subscales: closeness and conflict. Cronbach’s *α* for total score was 0.79 for current sample.

### Analyses

Descriptive statistics were used to characterised sample demographics (i.e. age, gender) and bereavement-related characteristics (i.e. relationship with deceased, time since loss).

Exploratory factor analysis of the Simpatico Scale was conducted using IBM SPSS Statistics version 27 for Windows (IBM Corp., Armonk, New York, USA; https://www.ibm.com/products/spss-statistics) to examine factor structure and scale reliability. A confirmatory factor analysis was conducted using IBM SPSS AMOS Statistics version 22 for Windows (IBM Corp., Armonk, New York, USA; https://www.ibm.com/products/structural-equation-modeling-sem) to assess the structural validity of the scale. Pearson correlations were used to determine convergent validity with the QRI-B and PG-13-R. Regression analyses were conducted to examine relationships between simpatico, demographic and bereavement-related characteristics, and social support variables (i.e. LSNS-6, ISEL-SF and INQ-15).

## Results

### Demographic and bereavement-related descriptive data

Table 1 summarises descriptive data of the full sample’s demographic and bereavement-related characteristics and their correlations and analysis of variance results with PGD (i.e. PG-13-R total scores). Additional exploratory analyses indicated that simpatico scores varied by relationship to the deceased (see Supplementary Table A2 available at https://doi.org/10.1192/bjo.2026.11028), whereas no significant differences were observed across cause-of-death categories (see Supplementary Table A3).

### Simpatico Scale psychometric results

#### Exploratory factor analysis of the Simpatico Scale

An exploratory factor analysis was conducted to explore the factor structure of the original 11-item Simpatico Scale, using data from 194 randomly selected participants. The sample consisted of 152 women and 42 men, and the mean age was 34.10 years (s.d. = 14.63). A principal component analysis with varimax rotation was conducted on 11 items.

An initial examination of the factor loadings of the 11-item Simpatico Scale revealed that the original Item 5 (‘Did you think that the deceased got you?’) exhibited cross-loading on 2 factors (0.68 and 0.46), whereas the original Item 9 (‘Did you think that you and the deceased had similar backgrounds?’) showed a very low loading on one factor (0.08) and had the lowest corrected item-total correlation (0.51). In addition to their limited statistical strength, these items were also considered semantically appropriate for removal. Accordingly, to improve factor structure clarity and scale reliability, they were excluded from the analysis. The revised analysis was conducted using the remaining nine items and showed excellent sampling adequacy (Kaiser–Meyer–Olkin measure of sampling adequacy 0.87, Bartlett’s test of sphericity *p* < 0.001) and a two-factor structure explaining 73.01% of the variance. The first factor, ‘felt connection,’ and the second, ‘felt similarities,’ were strongly correlated (*r* = 0.71, *p* < 0.001). A nine-item version of Simpatico Scale factor loadings results are shown in Table 2.

**Table 2:** Rotated component matrix of exploratory factor analysis of the nine-item Simpatico Scale (*n* = 194)

Simpatico item	Factor 1	Factor 2	Corrected item-total correlations	Communalities
	Felt connection	Felt similarities		
S1: Did you like the deceased?	**0.80**	0.21	0.69	0.70
S2: Did you feel a connection with the deceased?	**0.81**	0.31	0.74	0.74
S3: Did you feel like your true self when interacting with the deceased?	**0.84**	0.33	0.85	0.77
S4: Did you feel comfortable being yourself with the deceased?	**0.84**	0.28	0.82	0.71
S5: Did you see yourself in the deceased?	0.50	**0.66**	0.72	0.66
S6: Did you feel comfortable sharing vulnerabilities with the deceased?	0.44	**0.66**	0.68	0.59
S7: Did you think that you and the deceased had similar values?	0.33	**0.81**	0.79	0.73
S8: Did you think that you and the deceased had similar world views?	0.28	**0.82**	0.75	0.77
S9: Did you think that you and the deceased had similar world interests?	0.13	**0.83**	0.68	0.75
Cronbach’s *α*	0.86	0.88		
Spearman–Brown coefficient (equal length)	0.81	0.83		

Bolded statistics indicate key items per factor structure column. Original items 5 (‘Did you think that the deceased got you?’) and 9 (‘Did you think that you and the deceased had similar backgrounds?’) of the original Simpatico Scale^20^ were removed because of problematic factor loadings and low item-total correlations.

#### Confirmatory factor analysis of the Simpatico Scale

To validate the factor structure of the Simpatico Scale, which was finalised as a 9-item measure based on the results of the exploratory factor analysis, a confirmatory factor analysis was conducted on a randomly selected sample of 240 participants. The sample included 186 women (77.5%) and 54 men (22.5%), with a mean age of 33.99 years (s.d. = 14.23).

**Fig. 1:**
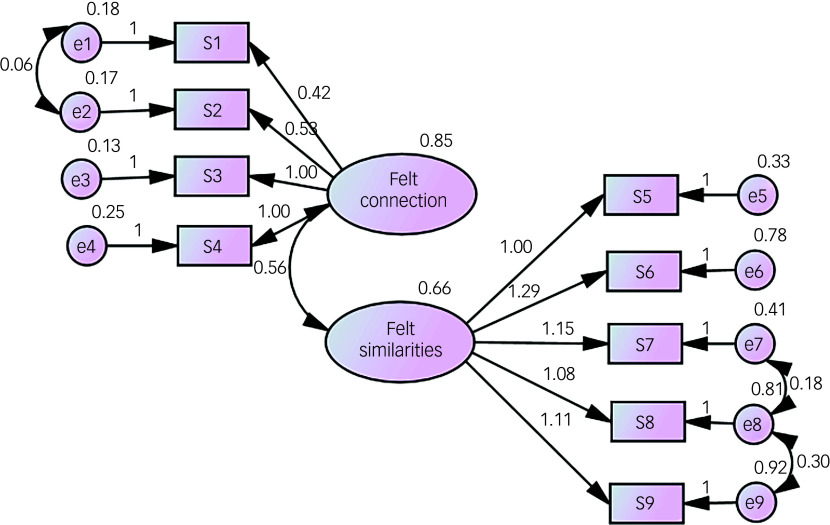
Confirmatory factor analysis results of the Turkish version of the Simpatico Scale (*n* = 240).

Following the high root mean square error of approximation (RMSEA) value (0.10) observed in the initial model, the proposed modifications were carefully reviewed. It was determined that, in addition to items 1 and 2, item 8 shared semantic overlap with items 7 and 9, all of which assess felt similarities with the deceased in terms of values, world views and interests. Accordingly, error covariances were added between these items, based on the rationale that participants’ responses to conceptually related items might be systematically associated. After adding three error covariances based on modification indices, which were also semantically similar, the model showed good fit: *χ* ^2^(29) = 57.32, *p* = 0.002, comparative fit index (CFI) 0.96, Tucker–Lewis index (TLI) 0.95, RMSEA = 0.07 (90% CI 0.04–0.09), standardised root mean square residual (SRMR) 0.04. Factor loadings were statistically significant (*p* < 0.001) and ranged from 0.67 to 0.93 (Fig. 1).

#### Convergent validity

Convergent validity of the Simpatico Scale was assessed through correlation analysis with QRI-B subscales. Results showed the Simpatico Scale had a strong positive correlation with QRI-B ‘Closeness’ (*r* = 0.77, *p* < 0.001) and a negative correlation with QRI-B ‘Conflict’ (*r* = −0.18, *p* < 0.001), supporting its convergent validity (see Supplementary Table A1 for full results).

#### Reliability

The Cronbach’s *α* coefficient was 0.86 for ‘felt connection’ (factor 1) and 0.88 for ‘felt similarities’ (factor 2), and 0.90 for the nine-item summary scale score, indicating high reliability. Additionally, the Spearman–Brown coefficients are 0.81 for factor 1, 0.83 for factor 2 and 0.83 for the total score, further supporting the high reliability of the scale (see Table 2).

### Grief-related results

#### Regression analyses

Serial linear regression models showed that ‘felt connection’ (factor 1) itself explained 9% of PG-13-R variance, whereas ‘felt similarities’ (factor 2) itself explained 20%. When two subscales entered the equation in model 3, ‘felt connection’ became non-significant; ‘felt similarities’ remained a significant predictor, accounting for 20% of the variance.

Model 4 included demographic, bereavement-related and social support variables. Model predictors were first examined for multicollinearity using bivariate analyses (see Table 3). Regression results showed that when demographic and loss-related factors were controlled in the first step (*R* ^2^ = 0.26, *F*(6, 392) = 24.64, *p* < 0.001); gender, deceased’s age, time since loss, relationship to deceased, cause of death, ‘felt similarities’ and INQ-15 ‘perceived burdensomeness’ to others after the loss were significant predictors of PG-13-R in the second step (*R* ^2^ = 0.44, *F*(15, 383) = 21.85, *p* < 0.001). See Table 3 for all regression model results.

**Table 3:** Regression analysis for the PG-13-R (*N* = 400)

Model	Predictor(s)	*B*	s.e.	*β*	*t*	*R* ^2^	*F*	Tolerance	VIF
Model 1	Simpatico 1 Felt connection	0.95	0.15	0.30	6.25***	0.09	39.03***	1.00	1.00
Model 2	Simpatico 2 Felt similarities	0.79	0.08	0.44	9.87***	0.20	97.494***	1.00	1.00
Model 3	Simpatico 1 Felt connection	0.01	0.19	0.01	0.06	0.19	48.63***	0.55	1.81
	Simpatico 2 Felt similarities	0.79	0.10	0.44	7.29***			0.55	1.81
Model 4 (step 2)	Gender^a^	1.75	0.85	0.08	2.06*	0.44	21.85***	0.901	1.10
	Age	−0.06	0.03	−0.09	−1.95			0.62	1.63
	Deceased’s age	−0.06	0.02	−0.15	−3.41***			0.77	1.29
	Time since loss	−0.05	0.01	−0.16	−4.07***			0.96	1.05
	Relationship to deceased person^b^	5.24	0.83	0.30	−6.28***			0.62	1.61
	Cause of death^c^	3.75	1.15	0.14	3.26**			0.80	1.26
	LSNS-6 Family	−0.13	0.13	−0.04	−0.96			0.74	1.35
	LSNS-6 Friend	−0.01	0.13	−0.01	−0.11			0.66	1.52
	ISEL-SF Appraisal	−0.41	0.18	−0.13	−2.29*			0.45	2.22
	ISEL-SF Belonging	−0.02	0.21	−0.01	−0.09			0.37	2.72
	ISEL-SF Tangible	−0.01	0.21	−0.01	−0.02			0.36	2.81
	INQ-15 Perceived burdensomeness	0.24	0.06	0.17	3.76***			0.72	1.39
	INQ-15 Thwarted belongingness	0.03	0.04	0.03	0.61			0.45	2.25
	Simpatico 1 (felt connection)	0.27	0.16	0.09	1.65			0.53	1.90
	Simpatico 2 (felt similarities)	0.54	0.10	0.31	5.76***			0.49	2.02

PG-13-R, Prolonged Grief-13-Revised; *B*, unstandardised regression coefficient; *β,* standardised coefficient; *t, t*-value testing the significance of each predictor; *R^2^*, proportion of variance in the outcome explained by the model; VIF, variance inflation factor; LSNS-6, Lubben Social Network Scale-6; ISEL-SF, Interpersonal Support Evaluation List-Short Form; INQ-15, Interpersonal Needs Questionnaire-15.

a. Gender was dummy coded as 1 = female and 0 = other.

b. Relationship to deceased person was dummy coded as 1 = first-degree relatives and 0 = non-first-degree relatives.

c. Cause of death was dummy coded as 1 = unnatural and 0 = natural causes.

*p < 0.05, **p < 0.01, ***p < 0.001.

**Fig. 2:**
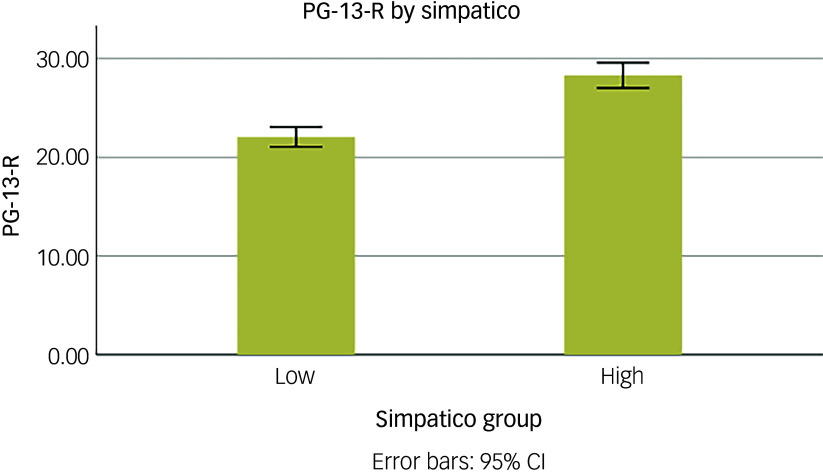
Prolonged grief disorder severity by high versus low simpatico relationship (median split). Groups were created using a median split (median: 39). PG-13-R, 13-item Prolonged Grief-13-Revised scale.

To further illustrate group differences, PGD severity was plotted by high versus low simpatico relationship, using a median split (see Fig. 2).

## Discussion

This study introduces the novel and micro-sociologically informed notion of simpatico (i.e. perceived interpersonal likeness and shared understanding) losses as risks for PGD. According to the micro-sociological theory of bereavement adjustment, losses which result in significant social voids and deprivations heighten PGD risk. Death of a simpatico other appears to be one such loss. In other words, people who survive the death of a simpatico other will experience the loss of someone who understood them and who held shared beliefs and values – a person who they perceived to be like themselves in essential ways. As a result, simpatico losses leave interpersonal voids in their wake, making mourners vulnerable to PGD. Additionally, simpatico may reflect the phenomenological experience of having felt deeply understood and connected to the deceased. The loss of such a bond may disrupt shared identity and meaning structures, intensifying grief responses not only through attachment separation, but also through the loss of relational self-continuity.

We developed the Simpatico Scale^21^ to measure this construct. In this report, we tested the scale’s validity and reliability in a bereaved adult sample based in Turkey. The scale revealed a two-factor structure: one factor consisting of ‘felt connection’, and another factor consisting of ‘felt similarities’ to the deceased person. The ‘felt connection’ factor, or subdimension, captures the mourner’s sense of authentic connection felt within the relationship and comfort in being their true self with the deceased. The ‘felt similarities’ factor, or subdimension, reflects the mourner’s degree of alignment with the deceased person, including perceived likeness, identification and shared values, perspectives and interests.

Confirmatory factor analysis indicated good model fit of the nine-item Simpatico Scale (*χ* ^2^/d.f. < 3, CFI and TLI > 0.90, RMSEA < 0.08, SRMR = 0.036).^29^ Convergent validity was supported by positive correlations with perceived closeness and divergent validity was supported by negative correlations with perceived conflict in the relationship with the deceased person. Both factors showed high internal consistency, with Cronbach’s reliability coefficients above 0.80, which is remarkable for factors/scales with five or fewer items.^30^

Our results reveal associations between simpatico scale scores, especially the similarities subdimension, and severity of PG-13-R scores. Notably, this association remained strong even after controlling for key demographic and bereavement-related and social support and relationship to the deceased factors known to relate to PGD. The findings suggest that the intensity of grief symptoms is linked to the fact that the deceased person was perceived as a simpatico figure who reflected the mourner’s sense of self, providing shared identity, meaning and mutual understanding. The loss of a simpatico relationship, a bond conceptualised for the first time in this study, may create a specific social void and lead to more severe grief reactions, as suggested by micro-sociological theory^1^ and supported by the cognitive attachment model of PGD, which posits that when a person’s identity is strongly entwined with the deceased, grief responses tend to be especially intense.^31^

Among various social support-related variables, perceived burdensomeness to others and guidance also emerged as significant predictors of PGD. Consistent with prior studies, perceiving oneself as a burden in relationships, and believing that one causes harm or makes others’ lives more difficult, contributes to the development of PGD during the grieving process.^32^ Importantly, these social support measures did not fully explain (mediate) the association between simpatico similarities with the deceased and PGD. Another important finding is that the absence of a reliable relationship where individuals can share their personal concerns and receive guidance is associated with an increase in PGD symptoms.^33^ This shows that the lack of relationships offering appraisal support (i.e. guidance, emotional understanding, making meaning) creates a critical social void during the grieving process, similar to – or possibly related to – the loss of simpatico bonds, which contributes to the development of PGD.

Recent meta-analyses have demonstrated the positive effects of social support on psychological symptoms, but have shown inconsistent results regarding grief symptoms.^34,35^ It appears that social variables should be examined with greater nuance. Our study contributes to filling this gap by identifying an interpersonal risk factor. This represents an important step toward understanding how variables offered by the concept of simpatico are related to socially relevant aspects of the grieving process.

Taken together, and especially by introducing the concept of simpatico, our study offers a novel perspective on grief across multiple social dimensions beyond social support. It is not the mere presence of support, but rather, the need for meaningful social bonds – where individuals feel recognised, understood, able to express their identity and share similarities, values and world views – that plays a critical role in the development of PGD, as suggested by micro-sociological theory.

### Clinical implications

Introducing the concept of simpatico not only offers a novel perspective on grief by addressing a distinct dimension of the social aspects of grief, but also identifies a specific intervention target. Support and peer groups^36^ and social-support based interventions (online or face to face)^37,38^ have been associated with grief-related outcomes or well-being. Recent literature also supports the need to have the bereaved person’s needs met by the support offered (e.g. if you need a friend to listen to you and they would prefer to drive you to a doctor’s office, your emotional needs will not be met by this practical form of social support).^39^ Given that simpatico is conceptualised as a similarity-based bond that is closely tied to the mourner’s sense of identity, continuity in close relationships and their way of making sense of the world, the findings suggest that maintaining, building or rebuilding meaningful simpatico-like relationships may offer support after loss.

Interventions that foster similarity-based interactions may be promising. As suggested by our findings, such interventions could aid in reconstructing sense of meaning,^40^ building meaningful social networks and restoring feelings of closeness and being understood. Further, such interventions may address the social void created by loss, thereby serving both supportive and protective functions in the grieving process.

### Limitations

Several limitations must be noted to best contextualise our findings and inform future promising avenues of research. First, the analysis includes results from a cross-sectional study; future longitudinal research is necessary to confirm the temporal nature of our PGD risk factor findings. Second, 17 participants were excluded from the study because they had previously received a psychiatric diagnosis (e.g. bipolar disorder, obsessive–compulsive disorder, etc.). Although the proportion (4.1%) is low, this exclusion may introduce a potential sample selection bias and may limit this study’s generalisability to bereaved persons with serious psychiatric illness. Third, the limited sample size and the restriction of the study to a specific geographic region can be considered as limitations. Additionally, no significant differences emerged between recruitment methods, likely because both approaches aimed to reach bereaved individuals from the general population. Future studies conducted with larger and more diverse samples, including subsamples based on relationship to the deceased or causes of loss, will not only help validate the Simpatico construct, but also contribute to a deeper understanding of its nature and relevance. Finally, the relationship between simpatico and the social void created by loss was beyond the scope of this study, and future explorations in this area will play an important role in advancing both research and interventions related to PGD.

In conclusion, our study introduces a novel interpersonal construct, simpatico, as a potential risk factor in the development of PGD. A nine-item Simpatico Scale was developed and psychometrically validated to measure this construct. Findings demonstrate that simpatico is a significant interpersonal predictor of PGD. Based on these results and the emerging literature, the construction of meaningful relationships rooted in felt similarity appears to be crucial in the grieving process. These insights may inform the design of more targeted grief interventions. In conclusion, this study identifies a valid and reliable construct derived from micro-sociological theory, offering new avenues for both future research and clinical practice in the field of grief.

## Supporting information

10.1192/bjo.2026.11028.sm001 Cesur-Soysal et al. supplementary material Cesur-Soysal et al. supplementary material

## Data Availability

The data that support the findings of this study are available from the corresponding author, G.C.-S., upon reasonable request.

## Supplementary material

The supplementary material is available online at https://doi.org/10.1192/bjo.2026.11028
