# Supplementary material for: A micro-sociological approach to prolonged grief disorder: identification and measurement of simpatico, a novel interpersonal risk factor
Source: BJPsych Open. 2026 Apr 24;12(3):e115. doi: 10.1192/bjo.2026.11028 (PMC13122337; doi:10.1192/bjo.2026.11028)
Supplement: Cesur-Soysal et al. supplementary material [file S205647242611028Xsup001.docx]

| **Appendix**  **Table A1.** |  |  |  |
| --- | --- | --- | --- |
| *Pearson correlations between the Simpatico scale and study variables* | | | |
| **Variable** | **Simpatico Scale** | | |
|  | Total | Felt Connection Subscale | Felt Similarities Subscale |
| Sex (female = 1)^a^ | -0.01 | -0.02 | -0.01 |
| Age | 0.12^*^ | 0.09 | 0.12^*^ |
| Deceased age | -0.20^***^ | -0.12^*^ | -0.23^***^ |
| Time since loss | 0.16** | 0.15** | 0.14** |
| Relationship to deceased (first-degree = 1)^b^ | 0.25*** | 0.17** | 0.26*** |
| Cause of death (unnatural = 1)^c^ | -0.07 | -0.05 | -0.07 |
| LSNS-6 Family | 0.14** | 0.18*** | 0.10* |
| LSNS-6 Friend | 0.04 | 0.07 | 0.02 |
| ISEL-SF Appraisal | 0.07 | 0.09 | 0.05 |
| ISEL-SF Belonging | 0.06 | 0.11* | 0.03 |
| ISEL-SF Tangible | 0.10* | 0.14** | 0.06 |
| INQ-15 Perceived Burdensomeness | 0.04 | -0.03 | 0.07 |
| INQ-15 Thwarted Belongingness | -0.18*** | -0.21*** | -0.14** |
| QRI-B Closeness | 0.77*** | 0.69*** | 0.72*** |
| QRI-B Conflict | -0.18*** | -0.25*** | -0.11* |
| PG-13-R | 0.43*** | 0.30** | 0.44** |
| **p* < .05, ***p* < .01, ****p* < .001  ^a^ Sex was dummy coded as 1 = female and 0 = other*.*  ^b^ Relationship to deceased person was dummy coded as 1 = first-degree relatives and 0 = non-first-degree relatives.  ^c^ Cause of death was dummy coded as 1 = unnatural and 0 = natural causes.  *Note.* PG-13-R = Prolonged Grief – 13 – Revised, QRI-B: The Quality of Relationships Inventory Bereavement Version, LSNS-6: The Lubben Social Network Scale-6, ISEL-SF: The Interpersonal Support Evaluation List-Short Form, INQ-15: The Interpersonal Needs Questionnaire-15, QRI-B: Quality of Relationships Inventory-Bereavement Version, SS: Simpatico Scale. | | | |

**Table A2.**

*Simpatico relationship scores by relationship to the deceased*

| **Relationship to the deceased** | **n** | **M** | **SD** | **Post Hoc (Games–Howell)** |
| --- | --- | --- | --- | --- |
| Mother | 57 | 39.40 | 6.28 | > Grandparent, > Second-degree relative |
| Father | 72 | 38.08 | 7.27 | - |
| Sibling / Brother / Sister | 20 | 40.45 | 3.46 | > Grandparent, > Second-degree relative |
| Spouse | 8 | 42.88 | 2.59 | > Father, > Grandparent, > Second-degree relative |
| Child | 11 | 40.45 | 4.70 | > Second-degree relative |
| Grandparent | 120 | 35.17 | 7.55 | < Mother, < Sibling, < Spouse, < Friend |
| Second-degree relative | 77 | 34.58 | 6.58 | < Mother, < Sibling, < Spouse, < Child, < Friend |

ANOVA. *F*(7, 392) = 6.84, *p* < .001, η² = .109.
Note. Homogeneity of variances was not met (Levene’s *p* = .005).

**Table A3.**

*Simpatico relationship scores by cause of death*

| **Cause of Death** | **n** | **M** | **SD** |
| --- | --- | --- | --- |
| Traffic accident | 25 | 36.76 | 8.52 |
| Accident (home, work, etc.) | 9 | 37.22 | 10.34 |
| Illness (sudden; e.g., heart attack, cancer) | 193 | 36.69 | 6.93 |
| Illness (expected/chronic) | 111 | 38.23 | 6.67 |
| Old age | 46 | 35.80 | 6.95 |
| Suicide | 11 | 39.00 | 4.02 |
| Natural disaster | 2 | 37.00 | 9.90 |

Note. One-way ANOVA indicated no significant differences across cause-of-death groups, *F*(7, 392) = 0.93, *p* = .480.
